# Supplementary material for: PHF13 is a molecular reader and transcriptional co-regulator of H3K4me2/3
Source: eLife. 2016 May 25;5:e10607. doi: 10.7554/eLife.10607 (PMC4915813; doi:10.7554/eLife.10607)
Supplement: Figure 3—source data 1. — DOI: http://dx.doi.org/10.7554/eLife.10607.006 [file elife-10607-fig3-data1.docx]

Figure 3 – supplement 1- source data 1: Data Collection and Refinement Statistics

| **Data collection and refinement statistics** | | |
| --- | --- | --- |
|  | apo-PHF13 | PHF13-H3K4me3 |
| **Data collection** |  |  |
| Space group | P4_3_2_1_2 | P2_1_2_1_2_1_ |
| Cell dimensions |  |  |
| *a*, *b*, *c* (Å) | 58.3, 58.315, 73.231 | 21.23, 44.33, 60.3 |
| α, β, γ (º) | 90, 90, 90 | 90, 90, 90 |
| Wavelength (Å) | 0.97904 | 0.97904 |
| Resolution (Å) | 50.0 – 1.85 (1.89 – 1.85) | 50.0 – 1.66 (1.70 – 1.66) |
| *R*_merge_ (%) | 5.4 (49.6) | 6.3 (32.2) |
| *I*/σ*I* | 58.0 (5.1) | 5.0 (24.7) |
| Completeness (%) | 99.9 (99.9) | 99.4 (91.0) |
| Redundancy | 15.5 (14.0) | 7.4 (5.8) |
|  |  |  |
| **Refinement** |  |  |
| Resolution (Å) | 27.38–1.85 | 35.73–1.67 |
| No. reflections | 10775 | 6772 |
| *R*_work_ / *R*_free_ | 18.4 / 18.8 | 18.3 / 22.4 |
| No. atoms |  |  |
| Protein | 443 | 416 |
| Zn^2+^ | 2 | 2 |
| Water | 41 | 60 |
| Peptide | 0 | 51 |
| B-factors (Å^2^) |  |  |
| Protein | 43.0 | 18.8 |
| Zn^2+^ | 36.8 | 12.5 |
| Water | 46.3 | 29.8 |
| Peptide | N/A | 23.0 |
| R.m.s. deviations |  |  |
| Bond lengths (Å) | 0.011 | 0.020 |
| Bond angles (º) | 1.215 | 1.586 |
| PDB code | 3O70 | 3O7A |
