# Supplementary material for: PHF13 is a molecular reader and transcriptional co-regulator of H3K4me2/3
Source: eLife. 2016 May 25;5:e10607. doi: 10.7554/eLife.10607 (PMC4915813; doi:10.7554/eLife.10607)
Supplement: Figure 3—source data 2. — (A) Binding of His-PHF13-PHD-only to a histone peptide array was detected with an anti-His antibody. Boxes denote the positive controls (12 x histidine) and an interaction with H3K4me3 when the neighboring R2 is not di-methylated. (B) Peptide key of the spotted differentially modified histone peptides on the histone peptide array. The length, position and sequence of the spotted peptides are annotated in the table. DOI: http://dx.doi.org/10.7554/eLife.10607.007 [file elife-10607-fig3-data2.docx]

Figure S3: Source data 2 - Interaction of PHF13’s PHD domain to differentially modified histone peptides

**A**

**B**


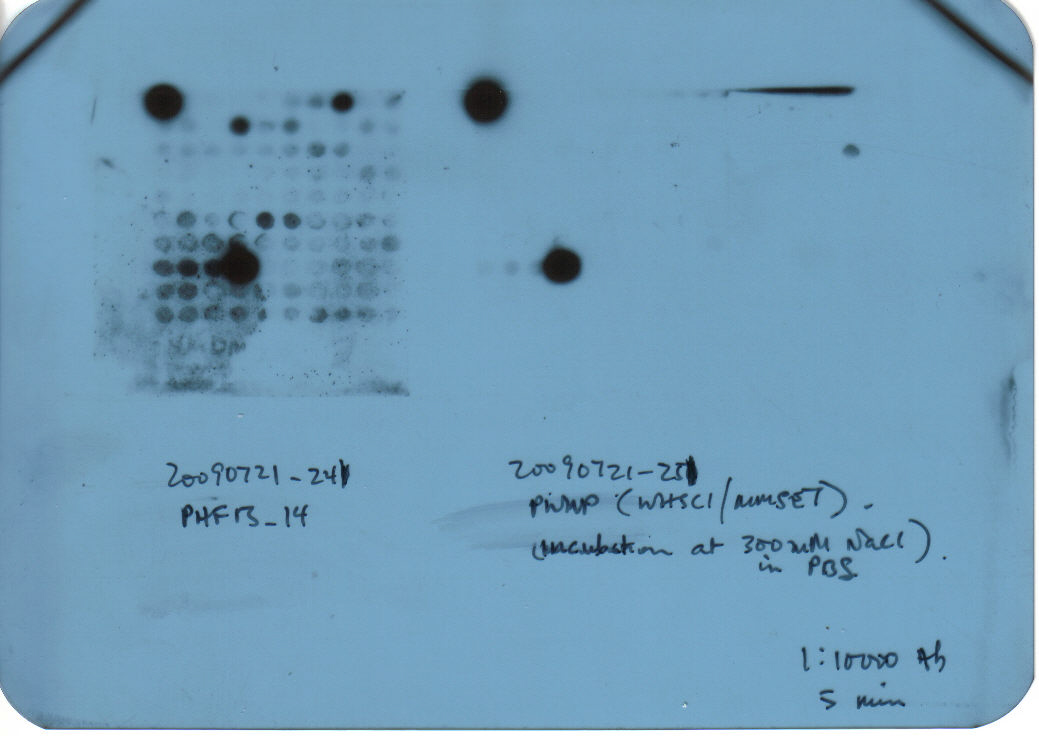


Peptide Grid # Modification Peptide Sequence

His (12x) A1 N/A HHHHHHHHHHHH

H3 (1-13) A2 ---- ARTKQTARKSTGG

H3 (1-13) A3 R2me ARmeTKQTARKSTGG

H3 (1-13) A4 R2me2s ARme2sTKQTARKSTGG

H3 (1-13) A5 R2me2a ARme2aTKQTARKSTGG

H3 (1-13) A6 K4me1 ARTKme1QTARKSTGG

H3 (1-13) A7 K4me2 ARTKme2QTARKSTGG

H3 (1-13) A8 K4me3 ARTKme3QTARKSTGG

H3 (1-13) A9 K9me1 ARTKQTARKme1STGG

H3 (1-13) A10 K9me2 ARTKQTARKme2STGG

H3 (1-13) B1 K9me3 ARTKQTARKme3STGG

H3 (1-13) B2 K9Ac ARTKQTARK_Ac_STGG

H3 (1-13) B3 S10ph ARTKQTARKS_p_TGG

H3 (1-13) B4 R2me/K4me3 ARmeTKme3QTARKSTGG

H3 (1-13) B5 R2me2/K4me3 ARmeTKme3QTARKSTGG

H3 (1-13) B6 R2me2a/K4me3 ARme2aTKme3QTARKSTGG

H3 (5-20) B7 ---- QTARKSTGGKAPRKQL

H3 (5-20) B8 S10p/K14Ac QTARKS_p_TGGK_Ac_APRKQL

H3 (5-20) B9 K14Ac QTARKSTGGK_Ac_APRKQL

H3 (5-20) B10 R17me1 QTARKSTGGKAPRme1KQL
