# Supplementary material for: PHF13 is a molecular reader and transcriptional co-regulator of H3K4me2/3
Source: eLife. 2016 May 25;5:e10607. doi: 10.7554/eLife.10607 (PMC4915813; doi:10.7554/eLife.10607)
Supplement: Figure 4—source data 1. — Table represents the proteins identified by mass spectrometry obtained from PHF13 immunoprecipitations from nuclease digested chromatin lysates from E14 ESCs that were either trypsin digested or LysC digested. Shown is the number of unique peptides identified as well as the posterior error probability (PEP) or q-value. DOI: http://dx.doi.org/10.7554/eLife.10607.010 [file elife-10607-fig4-data1.docx]

Figure 4 – figure supplement 1- source data 1: MS table of PHF13 combined interactions

| **Protein Names** | **Gene**  **Names** | **Unique**  **Peptides Cont.** | **Unique**  **Peptides PHF13** | **PEP** |
| --- | --- | --- | --- | --- |
| **Trypsin Digested** |  |  |  |  |
| Serine-protein kinase ATM | Atm | 0 | 23 | 4,8593E-269 |
| Transcription intermediary factor 1-beta | Trim28 | 3 | 13 | 1,03E-70 |
| DNA-directed RNA polymerase II subunit RPB1 | Polr2a | 0 | 10 | 1,7975E-167 |
| DNA-directed RNA polymerase II subunit RPB2 | Polr2b | 0 | 5 | 2,1498E-57 |
| Histone-binding protein RBBP4 | Rbbp4 | 0 | 4 | 3,2287E-267 |
| Splicing factor U2AF 35 kDa subunit | U2af1 | 0 | 3 | 2,11E-40 |
| Splicing factor 3B subunit 3 | Sf3b3 | 0 | 2 | 1,4307E-33 |
| Cell division cycle 5-like protein | Cdc5l | 0 | 2 | 9,6311E-25 |
| Pre-mRNA-splicing factor SPF27 | Bcas2 | 0 | 1 | 1,1592E-10 |
| Cleavage and polyadenylation specificity factor subunit 1 | Cpsf1 | 0 | 1 | 2,3588E-63 |
| Heterogeneous nuclear ribonucleoprotein R | Hnrnpr | 0 | 1 | 4,3496E-26 |
| Polyadenylate-binding protein 2 | Pabpn1 | 0 | 1 | 1,2539E-09 |
| PHD finger protein 13 | Phf13 | 0 | 1 | 0,000045104 |
| DNA-directed RNA polymerase II subunit RPB3 | Polr2c | 0 | 1 | 0,000053829 |
| DNA-directed RNA polymerases I, II, and III subunit RPABC3 | Polr2h | 0 | 1 | 8,7231E-13 |
| Histone-binding protein RBBP7 | Rbbp7 | 0 | 1 | 2,5399E-204 |
| U2 small nuclear ribonucleoprotein B | Snrpb2 | 0 | 1 | 0,000094054 |
| SNW domain-containing protein 1 | Snw1 | 0 | 1 | 5,674E-18 |
| **LysC Digested** |  |  |  | **Q-value** |
| DNA-directed RNA polymerase II subunit RPB1 | Polr2a | 0 | 4 | 0 |
| Heterogeneous nuclear ribonucleoprotein U | Hnrnpu | 0 | 4 | 0 |
| 116 kDa U5 small nuclear ribonucleoprotein component | Eftud2 | 0 | 3 | 0 |
| Splicing factor 3B subunit 1 | Sf3b1 | 0 | 3 | 0 |
| RNA binding motif protein, X chromosome;RNA-binding motif protein | Rbmx | 0 | 2 | 0 |
| Transcription intermediary factor 1-beta | Trim28 | 0 | 2 | 0 |
| Histone-binding protein RBBP4;RBBP7 | Rbbp4;Rbbp7 | 0 | 2 | 0 |
| Polycomb protein Suz12 | Suz12 | 0 | 1 | 0 |
| RNA binding motif protein, X-linked-like-1;RNA-binding motif protein, X chromosome | Rbmxl1 | 0 | 1 | 0.011628 |
| Heterogeneous nuclear ribonucleoprotein L | Hnrnpl | 0 | 1 | 0.00885 |
| Nuclease-sensitive element-binding protein 1;Y-box-binding protein 3 | Ybx1;Ybx3 | 0 | 1 | 0 |
| Heterogeneous nuclear ribonucleoprotein A3 | Hnrnpa3 | 0 | 1 | 0 |
| Pre-mRNA-processing factor 19 | Prpf19 | 0 | 1 | 0.009434 |
| Serine/arginine-rich splicing factor 5 | Srsf5 | 0 | 1 | 0 |
| RNA-binding proten FUS | Fus | 0 | 1 | 0 |
| Heterogeneous nuclear ribonucleoproteins A2/B1 | Hnrnpa2b1 | 0 | 1 | 0 |
| Serine/arginine-rich splicing factor 3 | Srsf5 | 0 | 1 | 0 |
| Splicing factor 3A subunit 1 | Sf3a1 | 0 | 1 | 0 |
| Pre-mRNA-processing factor 19 | Prpf6 | 0 | 1 | 0 |
| U4/U6.U5 tri-snRNP-associated protein 1 | Sart1 | 0 | 1 | 0 |
