# Supplementary material for: PHF13 is a molecular reader and transcriptional co-regulator of H3K4me2/3
Source: eLife. 2016 May 25;5:e10607. doi: 10.7554/eLife.10607 (PMC4915813; doi:10.7554/eLife.10607)
Supplement: Figure 4—source data 2. — Shown are the top biological processes and molecular functions and their false discovery rates (FDR), determined using by the string functional protein association network (http://string-db.org/) analysis. DOI: http://dx.doi.org/10.7554/eLife.10607.011 [file elife-10607-fig4-data2.docx]

Figure 4- figure supplement 4 - source data 2- Associated functional terms of PHF13 interacting proteins

| **Pathway description** | **FDR** |
| --- | --- |
| RNA metabolic process  **Biological**  **Processes** | 3.2e-20 |
| RNA processing | 7.69e-19 |
| gene expression | 1.14e-18 |
| nucleic acid metabolic process | 2.11e-18 |
| cellular aromatic compound metabolic process | 9.99e-18 |

| **Pathway description** | **FDR** |
| --- | --- |
| RNA binding | 2.19e-24 |
| poly(A) RNA binding  **Molecular**  **Function** | 5.3e-20 |
| nucleic acid binding | 1.62e-19 |
| heterocyclic compound binding | 5.13e-09 |
| organic cyclic compound binding | 6.17e-09 |
